# Supplementary material for: Campylobacter infection of young children in Colombia and its impact on the gastrointestinal environment
Source: mSphere. 2024 Sep 25;9(10):e00342-24. doi: 10.1128/msphere.00342-24 (PMC11520299; doi:10.1128/msphere.00342-24)
Supplement: Supplemental material — Figures S1-S5 and captions for supplemental tables. [file msphere.00342-24-s0001.pdf]

Figure S1. Microbiome 16S rRNA gene alpha diversity comparison across patient cohorts shows no significant difference using Kruskal-Wallis H tests in A.) observed ASVs (richness), B.) Pielou evenness, C.) Shannon diversity and D.) Faith phylogenetic distance (PD) metrics.

Figure S2. Microbiome 16S rRNA gene alpha diversity comparison between *Campylobacter* infected and uninfected patients shows no significant difference using Kruskal-Wallis H tests in A.) observed ASVs (richness), B.) Pielou evenness, C.) Shannon diversity and D.) Faith phylogenetic distance (PD) metrics.

Figure S3. Microbiome 16S rRNA gene alpha diversity comparison between symptomatic and asymptomatic patients shows no significant difference using Kruskal-Wallis H tests in A.) observed ASVs (richness), B.) Pielou evenness, C.) Shannon diversity and D.) Faith phylogenetic distance (PD) metrics.

Figure S4. Weighted UniFrac distances visualized on PCoA plots represent 16S rRNA gene community profiles are not significantly different by patient infection status, symptomatic status, and cohort as determined by adonis permutational multivariate analyses of variance models. Principal coordinate (PC) percentages represent the percent of total variation explained by that axis.

Figure S5. Metabolite beta diversity profiles were calculated with Canberra and Aitchison distances and visualized with t-SNE. Adonis permutational multivariate analyses of variance revealed a significant difference in Canberra distances between patient symptomatic status only, while Aitchison distances were only significantly different between infection status. Principal coordinate (PC) percentages represent the percent of total variation explained by that axis.

Table S1. ASV richness, Peilou's evenness, Shannon's diversity, and Faith's phylogenetic diversity statistics for the four cohorts, whether samples divided by Campylobacter infection status, or whether samples were obtained from symptomatic individuals.

Table S2. Weighted and unweighted UniFrac distances for the four cohorts, whether samples were divided by Campylobacter infection status, or whether samples were obtained from symptomatic individuals.

Table S3. ANCOM-BC measurements on which taxonomic classes were enriched or depleted in each cohort and whether those taxa were significantly differentially abundant.

Table S4. Peilou's evenness, Shannon's diversity, or richness of metabolome profiles for the four cohorts, whether samples were divided by Campylobacter infection status, or whether samples were obtained from symptomatic individuals.

Table S5. Permutational multivariate analyses of variance using Canberra distances or Aitchison distances.

Table S6. Random forest machine learning classifier model statistics that were used to determine if metabolite profiles were predictive of infection or symptomatic status.

Table S7. Microbe-metabolite abundance correlations that were used to explore the interactions and co-occurrences of microbial phyla and metabolites regardless of infection status, symptomatic status, or cohort.

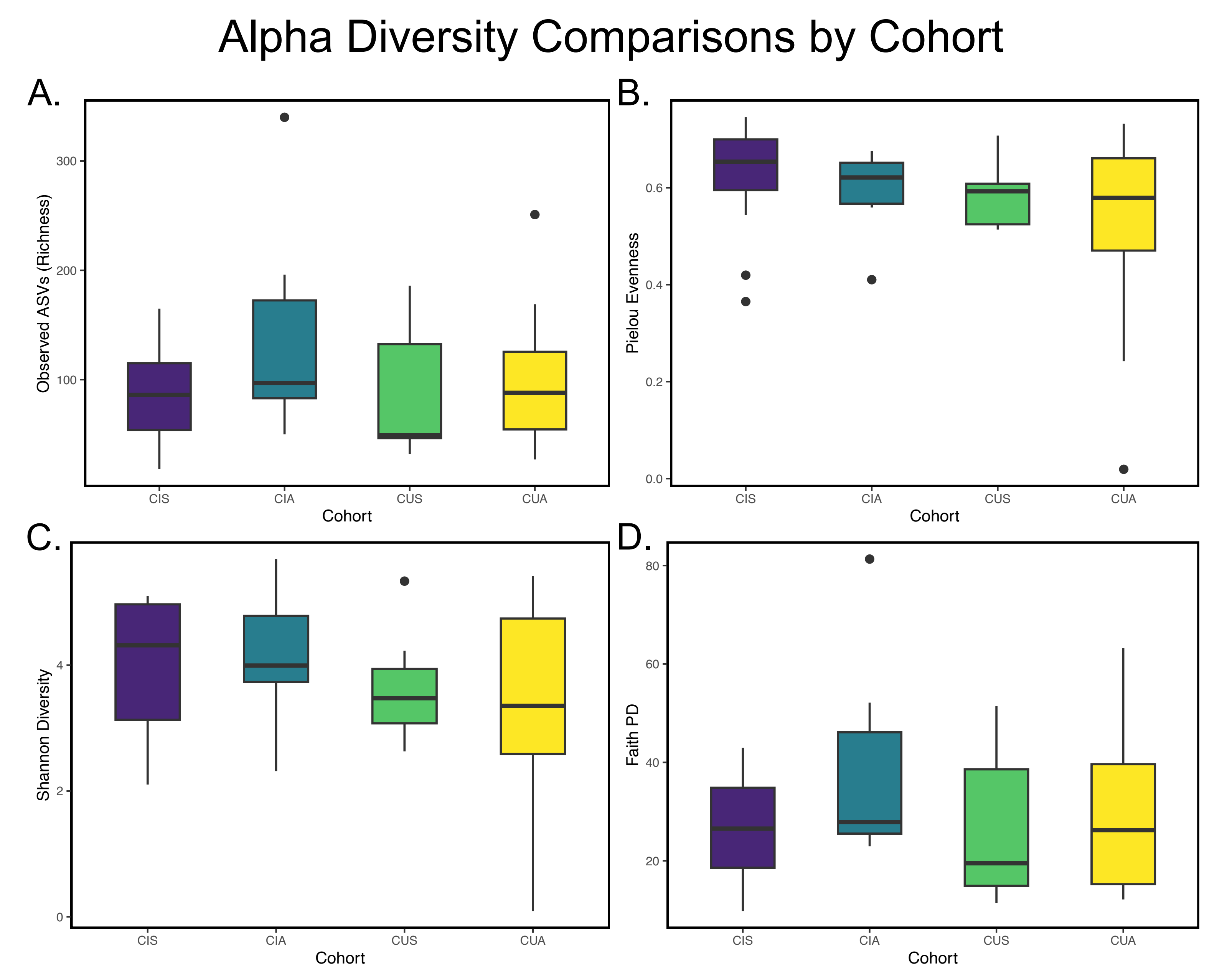

Alpha Diversity Comparisons by Cohort
A.
Observed ASVs (Richness)
300
200
100
CIS
CIA
CUS
CUA
Cohort
B.
Pielou Evenness
0.6
0.4
0.2
0.0
CIS
CIA
CUS
CUA
Cohort
C.
Shannon Diversity
4
2
0
CIS
CIA
CUS
CUA
Cohort
D.
Faith PD
80
60
40
20
CIS
CIA
CUS
CUA
Cohort

Alpha Diversity Comparisons by Infection Status

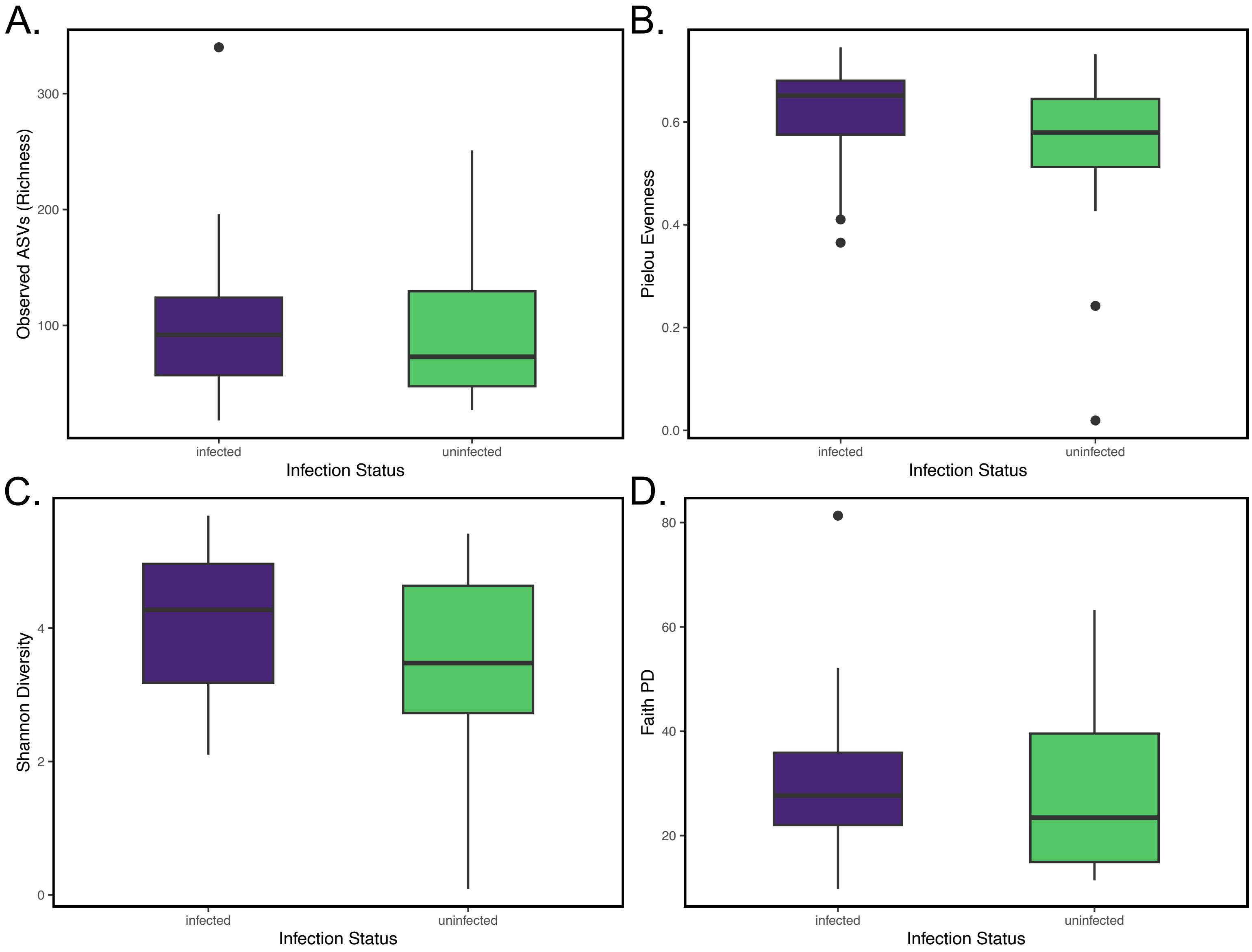

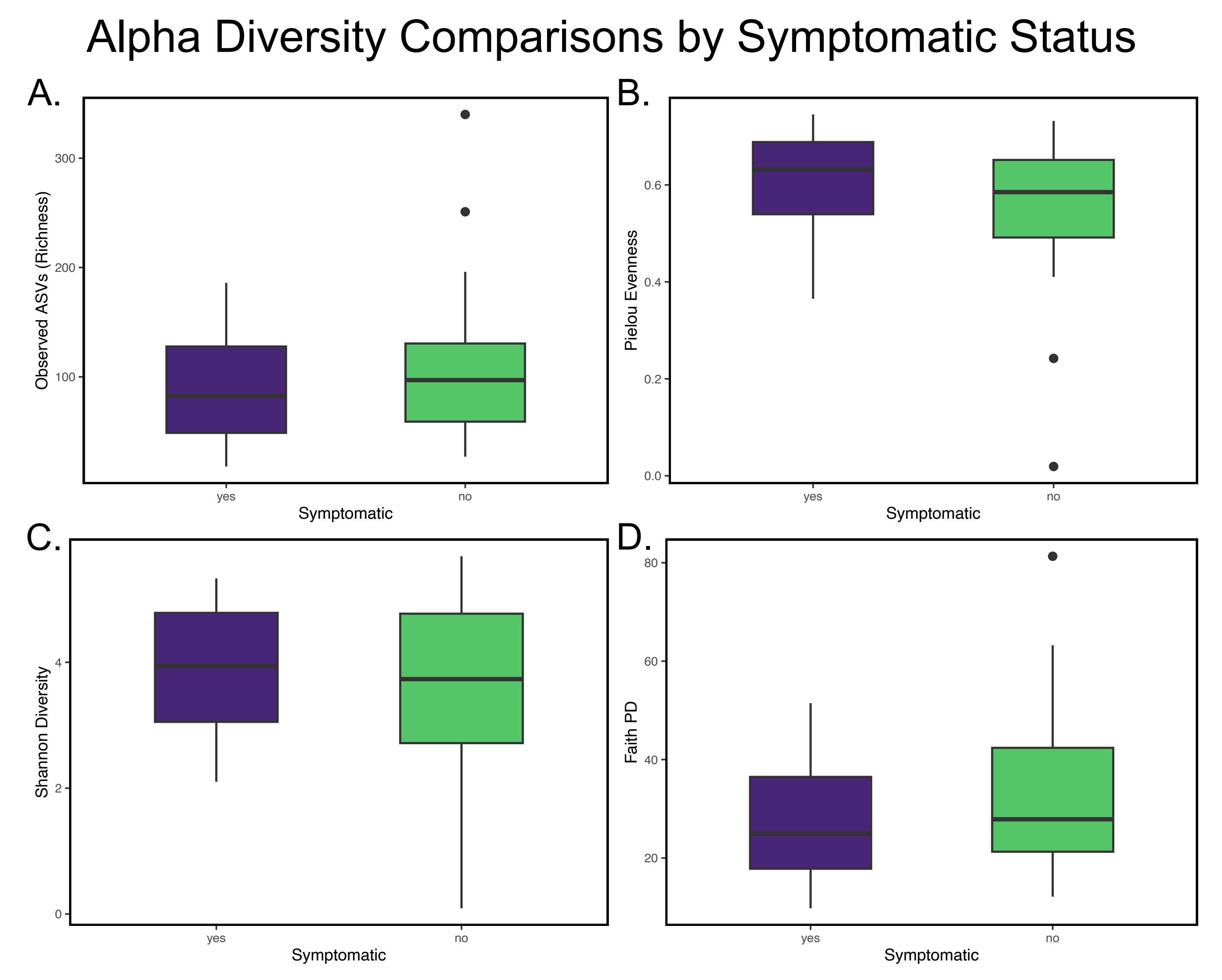

Alpha Diversity Comparisons by Symptomatic Status
A.
Observed ASVs (Richness)
300
200
100
yes
no
Symptomatic
B.
Pielou Evenness
0.6
0.4
0.2
0.0
yes
no
Symptomatic
C.
Shannon Diversity
4
2
0
yes
no
Symptomatic
D.
Faith PD
80
60
40
20
yes
no
Symptomatic

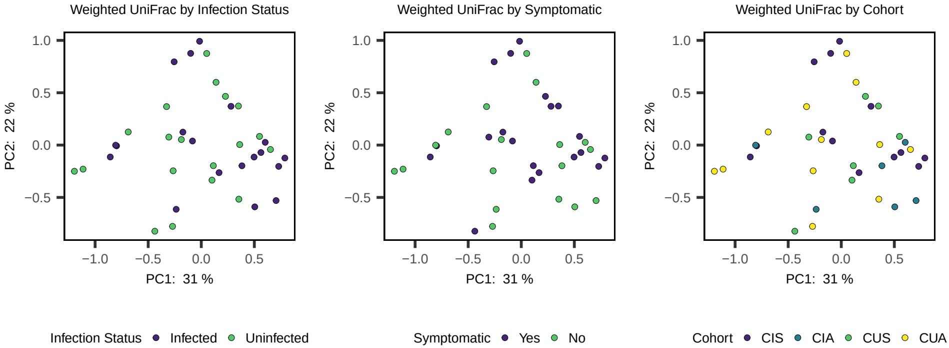
Weighted UniFrac by Infection Status
Weighted UniFrac by Symptomatic
Weighted UniFrac by Cohort
PC2: 22 %
PC1: 31 %
1.0
0.5
0.0
−0.5
−1.0
Infection Status
Infected
Uninfected
Symptomatic
Yes
No
Cohort
CIS
CIA
CUS
CUA

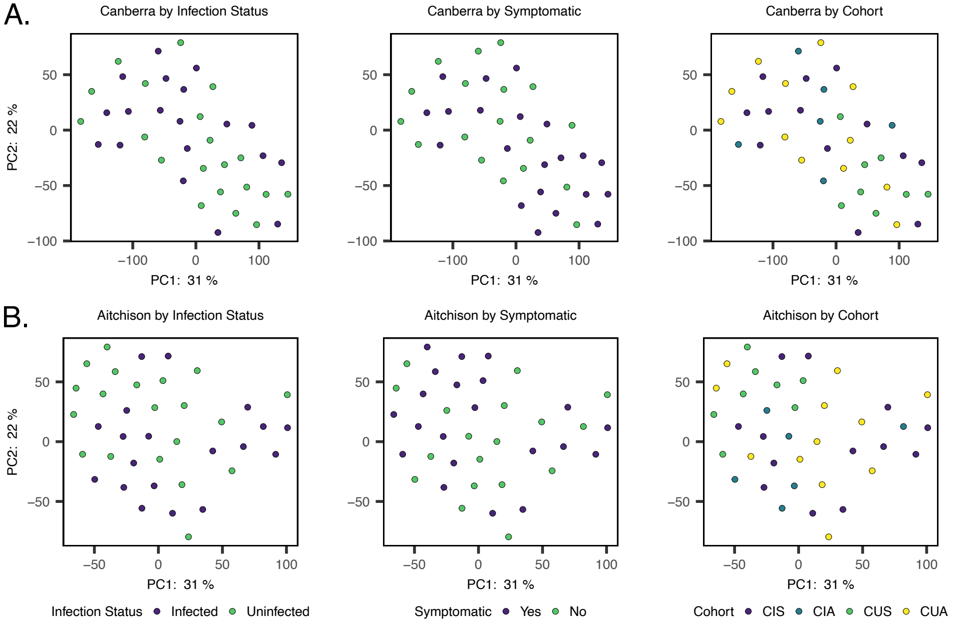
A.
Canberra by Infection Status
Canberra by Symptomatic
Canberra by Cohort
PC2: 22 %
PC1: 31 %
B.
Aitchison by Infection Status
Aitchison by Symptomatic
Aitchison by Cohort
PC2: 22 %
PC1: 31 %
Infection Status Infected Uninfected
Symptomatic Yes No
Cohort CIS CIA CUS CUA
